# Translating risk-based breast cancer screening to limited-resource settings

**DOI:** 10.1186/s44263-025-00176-4

**Published:** 2025-07-03

**Authors:** Kimberly Badal, Akash Maniam, Sarafina Urenna Otis, Laura J. Esserman

**Affiliations:** 1 Department of Surgery, University of California San Francisco, San Francisco, USA; 2 Caribbean Cancer Research Institute, El Socorro, Trinidad and Tobago; 3 Portsmouth Hospitals University NHS Trust, Portsmouth, UK; 4 Medical Sciences Division, University of Oxford, John Radcliffe Hospital, Oxford, UK

Risk-based breast cancer screening will likely become standard in high-resource settings if clinical trials show efficacy. Limited-resource settings will face significant barriers to implementation however an opportunity exists whereby risk-stratification could be used to optimize scarce resources by targeting women at highest risk, improving screening efficiency despite resource constraints.

## Background

Over 40 years ago, high-resource countries such as the USA, the UK, and Sweden implemented organized or opportunistic age-based breast cancer (BC) screening. Age-based screening was supported by evidence from clinical trials reporting a 20–30% mortality reduction in women invited to screen [1]. Since then, age-based BC screening has modestly reduced mortality but with substantial costs. For example, screening has not reduced the rate of advanced cancers over time, led to significant rates of overdiagnosis, and did not improve BC mortality rates after 10 years for women aged 40 to 49 years [2].

Further, mammography predominantly finds slower-growing BC subtypes (e.g., luminal A) while faster-growing subtypes (e.g., triple negative) are frequently found in the interval between screens [3]. Arguably, women who are at high risk for death due to diagnosis of faster-growing disease may gain the most from early detection, yet currently they do not. Another challenge is the lack of effective strategies for screening the 50–80% of women with heterogeneously or extremely dense breasts, where mammography is less sensitive. Finally, BC screening is resource intensive, costing $11 billion dollars in total direct costs in the USA in 2022 to screen approximately 37% of eligible women [4].

## The risk-based breast cancer screening paradigm

Acknowledging the flaws associated with a one-size-fits-all age-based breast cancer screening approach, a more advanced model of risk-based screening is being tested across several clinical trials globally. Here, risk assessment involves additional risk factors, such as family history, presence of pathogenic mutations, and polygenic risk scores to estimate a woman’s individual BC risk, which is then used to recommend a screening regimen. Clinical trials include the Women Informed to Screen Depending on Measures of risk (WISDOM) study in the USA [5]. If clinical trials report efficacy, personalized, risk-based screening will become the standard in high-resource settings. Unfortunately, no funding has been invested to investigate how this new paradigm will be translated to limited-resource settings (LRSs), which have the highest breast cancer mortality rates.

## Future challenges with implementing risk-based breast cancer screening in LRSs

In 2014, the World Health Organization (WHO) recommended against implementing age-based screening in LRSs since the criteria necessary for successful program implementation such as sufficient organizational and financial resources are unlikely to be met [6]. Despite this, policy makers in LRSs often receive pressure to implement BC screening with the assumption that the benefits reported in high-resource settings would translate to LRSs. As anticipated, opportunistic mammography screening programs in some LRSs have not demonstrated benefit in reducing BC mortality [7]. However, screening with clinical breast exams seems to be a feasible and cost-effective alternative to lower the stage at diagnosis in LRSs, though it is unknown whether it reduces BC mortality [7].

The challenges associated with implementing age-based screening in LRSs outlined by the WHO 2014 will still apply to risk-based screening. In addition to these challenges, risk-based screening will bring new challenges, specifically: (i) under-specification of the existing risk models to the risk factor and disease epidemiology of LRSs; and (ii) higher inability to implement the more complex and resource-intensive risk assessment and screening strategies recommended. In other words, the barrier to uptake of risk-based screening in LRSs will likely be higher than for age-based screening.

Current BC risk models have poorer discrimination for faster-growing human epidermal growth factor 2 receptor and triple negative subtypes [3], which often are more prevalent in LRSs compared to high-resource settings [8]. Further, the most important BC risk factors that need to be considered during risk assessment will likely differ within and between LRSs. For example, populations with higher exposure to environmental risk factors such as air, water, and food pollutants, or populations who experience social risk factors, such as poverty, may have their BC risk underestimated if these factors are not considered. There is also a lack of requisite research expertise and support in LRSs to develop local risk models.

Feasibly implementing risk-based screening will require the availability of reliable data inputs for risk assessment and the recommended screening and risk-reducing interventions. Many health systems in LRSs will not have access to the complex data inputs required for risk assessment such as polygenic risk scores or have access to screening mammography or magnetic resonance imaging (MRI) due to lack of availability and financial constraints. MRI, which is recommended yearly in women at highest risk by the American Cancer Society [9] and American College of Radiology [10], is especially inaccessible for screening in LRSs due to the high cost for purchase and maintenance by the health system. Risk can also be more accurately assessed using deep learning models that read mammogram images [11]. This strategy is arguably less accessible to LRSs as many do not have digital mammograms, technical infrastructure or human resources needed to implement artificial intelligence-based risk assessment. Lastly, the practice of personalized medicine is not commonplace in many LRSs; therefore, other barriers to implementation such as lack of awareness and trust will also need to be addressed.

## Opportunities for risk-based breast cancer screening to benefit LRSs

Despite the challenges, risk-based screening presents a compelling opportunity to optimize the allocation of scarce screening resources in LRS. By effectively identifying women at the highest risk, financial and technical resources can be strategically targeted for interventions. However, as previously discussed, a critical barrier lies in the development of risk models that are applicable to the local population and feasible to implement. One solution involves testing validated, publicly available risk models requiring minimal, readily obtainable data points in the target population such as the Breast Cancer Surveillance Consortium risk models [12]. Another solution is that researchers could explore alternative methods for identifying high-risk subgroups other than the use of risk models. This might entail identifying specific communities, geographic regions, or occupational groups known or suspected to have a higher breast cancer burden.

The application of risk models at the population level suggests that the proportion of women categorized as high-risk is likely to be relatively small (1–13%) [5], which makes selectively targeting these populations with available resources a potentially feasible and cost-effective approach in LRS. Feasible interventions for women at highest risk may include educational campaigns to raise awareness, screening with clinical breast exams or mammography, and promoting low-cost risk-reduction strategies like dietary and lifestyle modifications. The specific screening modality employed will depend on the available resources. Conversely, women classified as lower risk could receive less frequent interventions or potentially forego any intervention altogether. Evaluation of the feasibility, efficacy, and ethical considerations associated with selectively targeting high-risk women would also be needed.

Given that implementing a risk-based screening trial in LRSs may be infeasible for study teams due to lack of funding and competing priorities, we highlight steps that can be taken by these teams to maximize future benefit to LRSs (Table 1).

**Table 1:** Recommendations for risk-based screening trials teams to ensure that trial results can benefit populations in LRSs

Recommendation	Rationale
1. Expand screening study to local underserved communities	Women in local underserved communities likely have similar constraints in access, exposure to social and environmental risk factors, and epidemiology of disease as women in LRSs
2. Develop and test models that predict subtype-specific breast cancer risk	Women in LRSs have been shown to have higher prevalence of faster-growing BC subtypes than women in high-resource settings
3. Integrate non-biological drivers of risk in risk models	Social and environmental risk factors that likely contribute to the burden of disease in LRSs not presently considered by risk models
4. Test low-cost screening (e.g., clinical breast exam) and risk-reducing interventions (e.g., lifestyle changes) alongside state-of-the-art interventions	Capacity to benefit from risk-based screening will be undermined by lack of access to recommended interventions
5. Include researchers from LRSs in study teams	Researchers from LRSs will be positioned to advise on the challenges and opportunities associated with risk-based screening for their setting

## Conclusions

Risk-based screening offers an opportunity to determine whether focusing screening resources on women at highest risk in LRSs is an effective, alternative approach to population-based screening, which is not recommended for LRSs. Different strategies will need to be tested to identify women at highest risk in LRSs and researchers testing risk-based screening in clinical trials should take steps to maximize the transportability of the results to LRSs.

## Data Availability

No datasets were generated or analysed during the current study.

## Abbreviations

USA: United States of America
BC: Breast cancer
LRSs: Limited-resource settings
WISDOM: Women Informed to Screen Depending on Measures of risk
MRI: Magnetic resonance imaging
